# The morphological characteristics of hippocampus and thalamus in mesial temporal lobe epilepsy

**DOI:** 10.1186/s12883-020-01817-x

**Published:** 2020-06-08

**Authors:** Dongyan Wu, Feiyan Chang, Dantao Peng, Sheng Xie, Xiaoxuan Li, Wenjing Zheng

**Affiliations:** 1 Department of Neurology, China-Japan Friendship Hospital, Beijing, 100029 China; 2 Department of Radiology, China-Japan Friendship Hospital, Beijing, 100029 China

**Keywords:** Hippocampus, Thalamus, Morphology, Temporal lobe epilepsy

## Abstract

**Background:**

Mesial temporal lobe epilepsy (MTLE) is the most common form of focal epilepsy, which is frequently characterized by hippocampal sclerosis (HS). Accumulating studies have suggested widespread cortico-cortical connections related to MTLE. The role of subcortical structures involved in general epilepsy has been extensively investigated, but it is still limited in MTLE. Our purpose was to determine the specific morphological correlation between sclerotic hippocampal and thalamic sub-regions, using quantitative analysis, in MTLE.

**Methods:**

In this study, 23 MTLE patients with unilateral hippocampal sclerosis and 24 healthy controls were examined with three-dimensional T1 MRI. Volume quantitative analysis in the hippocampus and thalamus was conducted and group-related volumetric difference was assessed. Moreover, vertex analysis was further performed using automated software to delineate detailed morphological patterns of the hippocampus and thalamus. The correlation was used to examine whether there is a relationship between volume changes of two subcortical structures and clinical characteristics.

**Results:**

The patients had a significant volume decrease in the sclerotic hippocampus (*p* < 0.001). Compared to controls, obvious atrophic patterns were observed in the bilateral hippocampus in MTLE (*p* < 0.05). Only small patches of shrinkage were noted in the bilateral thalamus (*p* < 0.05). Moreover, the volume change of the hippocampus had a significant positive correlation with that of the thalamus (*P* < 0.001). Intriguingly, volume changes of the hippocampus and thalamus were correlated with the duration of epilepsy (hippocampus: *P* = 0.024; thalamus: *P* = 0.022). However, only volume changes of thalamus possibly differentiated between two prognostic groups in patients (*P* = 0.026).

**Conclusions:**

We demonstrated the morphological characteristics of the hippocampus and thalamus in MTLE, providing new insights into the interrelated mechanisms between the hippocampus and thalamus, which have potential clinical significance for refining neuromodulated targets.

## Background

Mesial temporal lobe epilepsy (MTLE) is the most common form of focal epilepsy. Currently, MTLE with hippocampal sclerosis (HS) continues to be of great interest to researchers because it has high prevalence and a relatively uniform clinical symptom of epileptic seizure. The morphological hallmark of the epileptogenic hippocampus is atrophy in MTLE [1, 2], which can be detected clinically on MRI. Accumulating studies have revealed that multiple extra-temporal cortexes, such as the frontal, insular, and even the parietal lobes are affected [2–8], suggesting widespread cortico-cortical connections related to MTLE.

The cortico-subcortical circuit of epilepsy has been known for more than half a century. The thalamus is, in particular, emphasized because of its diffused reciprocal interconnection with the cortex physiologically [9]. The role of the thalamus in generalized seizures has been extensively studied, but interest has also been sparked to address the relationship between the thalamus and focal seizures in recent years. The functional alteration of the thalamus in the context of MTLE has been described [10–16], implying the involvement of hippocampal-thalamic loops in MTLE. Whether the shape and volume of the thalamus are affected in patients with MTLE, however, is still unknown.

Based on quantitative analysis, we investigated volumetric alteration and shape pattern of sclerotic hippocampus and thalamus in 23 patients with MTLE and 24 controls, demonstrating the sub-region dependent relationship between these two subcortical structures, which has potential clinical significance. Then we further identified, through analyzing the correlation, the relationship between volume alteration of the hippocampus or thalamus and clinical characteristics. Delineation of the morphological correlation between the sclerotic hippocampus and thalamus would further confirm the influence on subcortical structure and deepen our understanding of the mechanisms in MTLE.

## Methods

### Patients

In total, 23 patients (14 men, mean ± standard deviation (SD) age = 26.09 ± 6.30 years) were retrospectively included in this study from 2010.6 to 2015.12 in the China-Japan Friendship Hospital, Beijing, China. All of the patients were diagnosed with MTLE with unilateral HS by a comprehensive clinical history, seizure semiology, scalp EEG, and MRI. 14 patients had hippocampal sclerosis on the left side, while the others were on the right side according to the diagnostic criteria of brain MRI [2]. In addition, 24 controls (15 men, mean ± SD age = 26.29 ± 4.62 years) matching for age and sex were included in our study.

Resective surgery was carried out on 19 patients, and postoperative seizure outcomes were measured during the follow-up, which were assessed using Engel’s classification; 12 (63%) patients had a class I, 4 (21%) a class II, 2 (11%) a class III, and 1 (5%) a class IV outcome. For analysis, Grades I and II were considered to be favorable surgical outcomes, whereas Grades III and IV were considered poor outcomes. Otherwise, the seizure outcome was described individually.

The China-Japan Friendship Hospital Research Ethics Committee approved the study and written informed consent was obtained from all participants.

### MRI scanning

All patients and controls underwent MRI on a 3 Tesla scanner (GE Medical Systems, USA). The following sequences were performed, including a T1-weighted 3D gradient-echo sequence (TR = 6.40 ms, TI = 400 ms, TE = 2.80 ms, flip angle = 20°, matrix = 256 × 256, voxel size = 1 mm × 1 mm × 1 mm) and conventional MR imaging (standard coronal T2-weighted sequences, fluid-attenuated inversion recovery (FLAIR) images).

### Image processing

MR images of the patients with right-sided MTLE were flipped onto the left side, allowing for the study of a ‘single’ group with an epileptic focus uniformly lateralized to the left.

#### Volumetric measurement

**Fig. 1:**
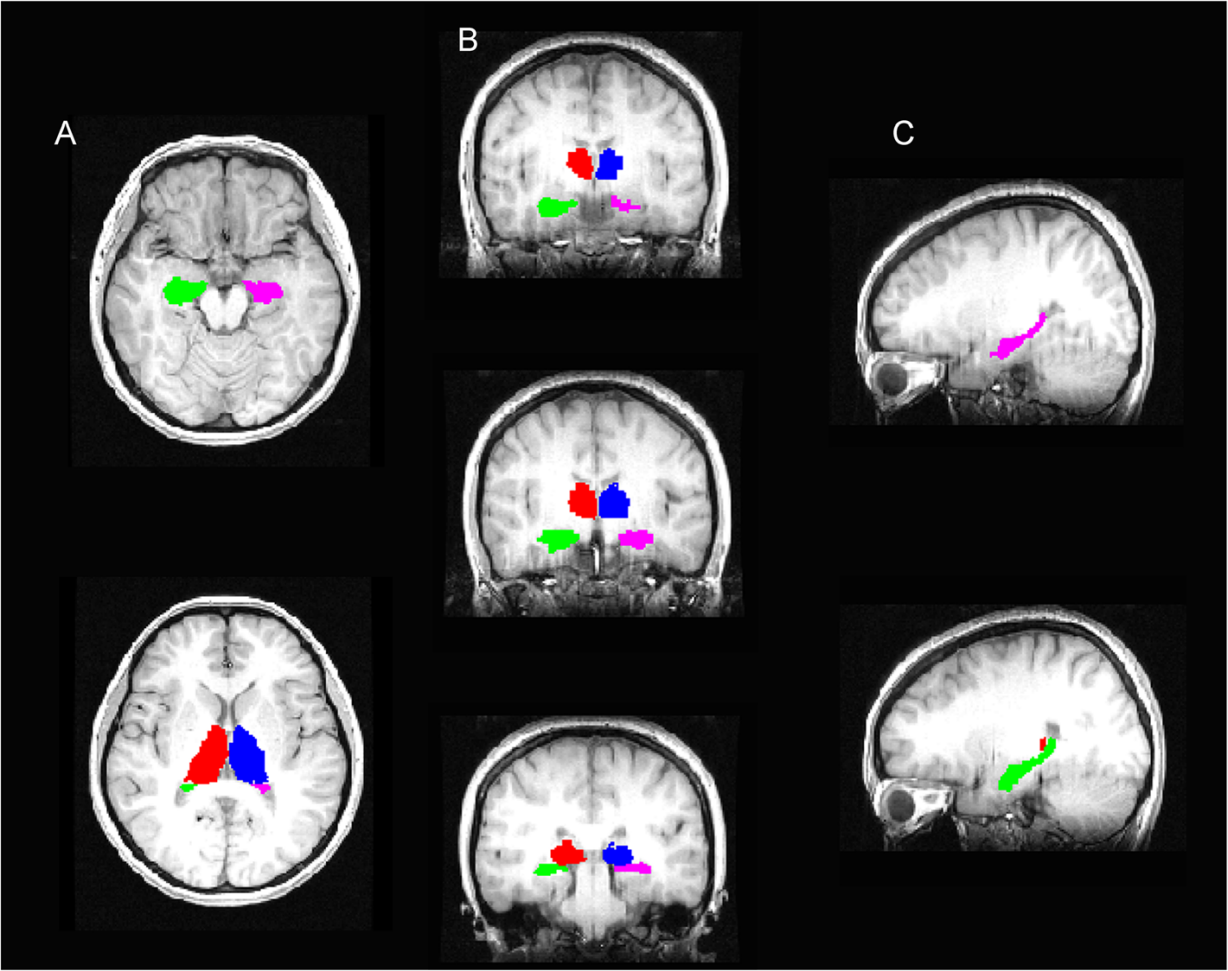
The segmented hippocampus and thalamus are shown on the patient’s MRI. A: The segmented hippocampus and thalamus are in a transverse section. B: From the anterior to posterior view, the segmented hippocampus and thalamus are in a coronal section. C: The bilateral hippocampi are segmented in a sagittal section (HS lateral, up; Contralateral, down)

In this study, we segmented bilateral volumes of hippocampus and thalamus on the basis of the T1-weighted 3D image using FreeSurfer software package (version 6.0. http://surfer.nmr.mgh.harvard.edu/) (Fig. 1). In a previous study, this method was applied to analyze the volume of subcortical structure and has been described extensively elsewhere [17]. Briefly, volume data for each subject were extracted from images acquired during the standard workflow, including co-registering the image into MNI305 space, stripping skull, automatically segmenting, parcellating, and assigning a neuroanatomical label to subcortical volumes. In addition to obtaining bilateral hippocampal and thalamic volumes, intracranial volume was also measured. Hippocampal and thalamic segmentations were visually inspected to confirm they were correct to avoid segmentation errors in all subjects.

Bilateral hippocampal and thalamic volumes were further normalized by dividing intracranial volume individually.

#### Vertex-wise analysis

We used FIRST software (version 5.0) (FMRIB’s Software Library, a part of the FSL software package-version 5.0, http://www.fmrib.ox.ac.uk/fsl) to perform vertex-wise analysis of the hippocampus and thalamus.

This approach has been described in previous studies [11, 17, 18]. The shape information about the hippocampus and thalamus of each subject was extracted from the 3D T1-weighted MR images. Deformable surfaces of two subcortical structures were used to automatically parameterize the volumetric labels in terms of meshes. The surface meshes of segmented structures were constructed in the native space of the image and then registered to the Montreal Neurological Institute space. The permutation test was applied to determine whether group comparisons of vertices were significantly smaller in patients with MTLE than in controls with the T statistics. Specifically, the above procedure was re-applied 1000 times.

### Statistical analysis

Demographic and clinical characteristics were performed using two-tailed Student’s t-tests or Chi-squared tests. The data were tested for a normal distribution before implementing parametric analysis. Subsequently, group-related normalized volumes of the hippocampus and thalamus were performed by two-tailed independent-samples t-test. Pearson’s correlations were used to investigate relationships between a percent change of the sclerotic hippocampus and the ipsilateral thalamus (a percent change of hippocampus: (epileptogenic-contralateral)/contralateral volumes, a percent change of thalamus: (epileptogenic-contralateral)/contralateral volumes) and clinical variables in patients. For comparisons of volume changes in these two subcortical structures between patients who were postoperatively found to have a favourable outcome and patients who continued to experience persistent postoperative seizures, a univariate ANOVA was used, including two prognostic groups with factor and volume changes (hippocampus, thalamus) as dependent variables. The statistical analyses were performed using SPSS software (IBM SPSS Statistics, Version 24.0; Armonk, NY, USA) for Windows. Statistical significance was defined as *p* < 0.05 after Bonferroni correction.

## Results

### Clinical characteristics

The demographic data and clinical characteristics of enrolled patients are summarized in Table 1. In the MTLE group, the mean age of seizure onset was from 0.5 to 29 years (13.33 ± 7.60 years) and the mean duration of epilepsy was 12.52 ± 6.95 years. The patients had different frequencies of clinical seizures, and the average seizure frequency was 3.44 ± 2.92 (range = 1–12) per month.

**Table 1:** Clinical characteristics of MTLE patients

Patient	Onset	Duration (years)	Febrile Seizures	Type	Onset zone of ictal discharges	MRI	Surgical procedure	Surgical outcome#
1	9	19	+	ET + ATM + BTC	Right	Right HS	ATL	IV
2	17	2	–	AN+ATM + BTC	Left	Left HS	ATL	I
3	11	16	–	ET + ATM	Right	Right HS	ATL	III
4	9	8	–	AN+ATM + BTC	Left	Left HS	ATL	I
5	17	15	–	ATM	Right	Right HS	ATL	II
6	0.5	18	–	AN+ATM + BTC	Left	Left HS	ATL	I
7	15	12	+	AN+BA +BTC	Left	Left HS	ATL	I
8	7	19	–	ATM + BA	Right	Right HS	ATL	I
9	14	7	–	ET + AN+ATM	Right	Right HS	ATL	I
10	1	20	–	AN+ATM	Left	Left HS	ATL	I
11	11	12	+	BA	Left	Left HS	ATL	II
12	15	12	+	AN+ATM +BTC	Right	Right HS	ATL	II
13	2	34	–	CN + ATM +BTC	Left	Left HS	ATL	I
14	18	10	+	BA+BTC	Right	Right HS	ATL	III
15	17	11	–	AN+ET + BTC	Left	Left HS	ATL	I
16	9	18	–	BA+BTC	Left	Left HS	–	–
17	19	7	–	AN+BTC	Left	Left HS	ATL	I
18	14	13	–	AN+ATM	Left	Left HS	ATL	II
19	28	8	–	AN+BA+BTC	Right	Right HS	ATL	I
20	26	11	–	AN+ATM	Right	Right HS	ATL	I
21	12	6	–	AN+ATM +BTC	Left	Left HS	–	–
22	12	4	–	ATM	Left	Left HS	–	–
23	29	6	–	AN++SN + BTC	Left	Left HS	–	–

Onset: onset of seizures; ET: emotional seizure; ATM: automatisms; BA: behavior arrest; CN: cognitive seizure; AN: autonomic seizure; BTC: bilateral tonic-clonic seizures; Left: left anterior temporal lobe; Right: right anterior temporal lobe; HS: hippocampal sclerosis; ATL: standard anterior temporal lobectomy; #: According to the Engel’s classification and the ILAE outcome classification, Grades I and II were considered as favorable surgical outcome, whereas Grades III and IV were considered poor; −: The patients had not epilepsy surgery.

### MRI characteristics

Conventional MR images were examined for the presence of unilateral hippocampal atrophy in all patients. Moreover, loss of internal architecture and increased T2 signals were only revealed in the sclerotic hippocampus. However, the contralateral hippocampus was normal. In contrast, the thalamus did not show any structural or signal changes upon visual inspection.

### Quantitative volumetric data

**Fig. 2:**
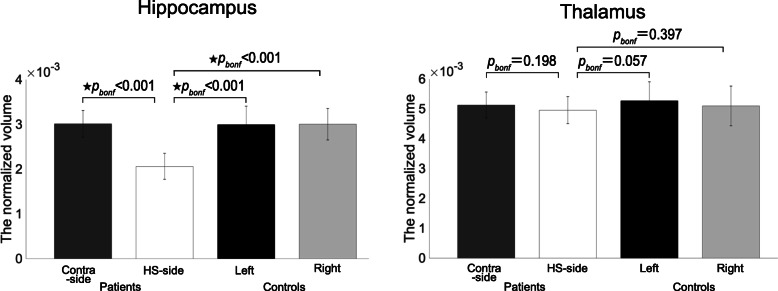
Hippocampal and thalamic volumes in patients with MTLE and controls. Group comparisons of the normalized volumes of hippocampus and thalamus in two groups. The black star () shows statistically significant difference

The normalized volumes of the hippocampus and thalamus were compared, and the results are shown in Fig. 2. In the patient group, a prominent volume decrease was detected in the sclerotic hippocampus, however we detected no significant reduction in thalamic volume (*p*_*bon*f_ < 0.05). In the control group, no volume difference was found between the bilateral hippocampus and thalamus (*p*_*bonf*_ < 0.05).

### Vertex analysis

**Fig. 3:**
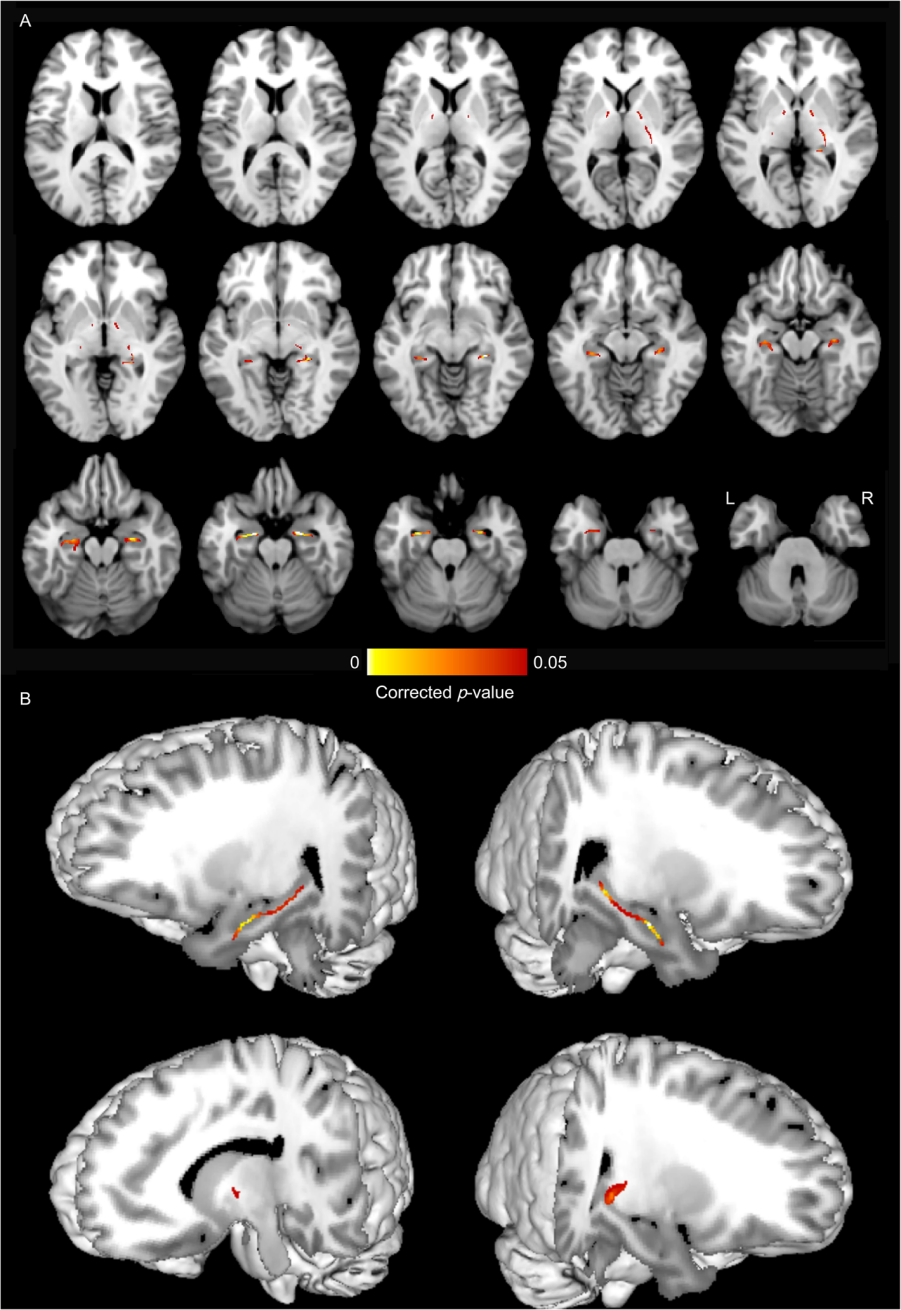
Focal atrophy of hippocampus and thalamus in MTLE. Shape comparison of hippocampus and thalamus between patients with MTLE and controls show focal atrophy in bilateral hippocampus and thalamus. Focal atrophy of hippocampus and thalamus with the T statistics are displayed at each vertex (p_perm_ < 0.05). Both (A) Horizontal view and (B) 3D view were displayed

Significant regional contractions were mainly located in the bilateral hippocampus in MTLE group (Fig. 3, *p*_*perm*_ < 0.05)). In particular, significant atrophy was shown in the hippocampal head on the ipsilateral side with HS. Only small patches of shrinkage were noted in the bilateral thalamus (Fig. 3). The hippocampus and thalamus did not show any local expansion.

### Correlations and clinical variables

**Fig. 4:**
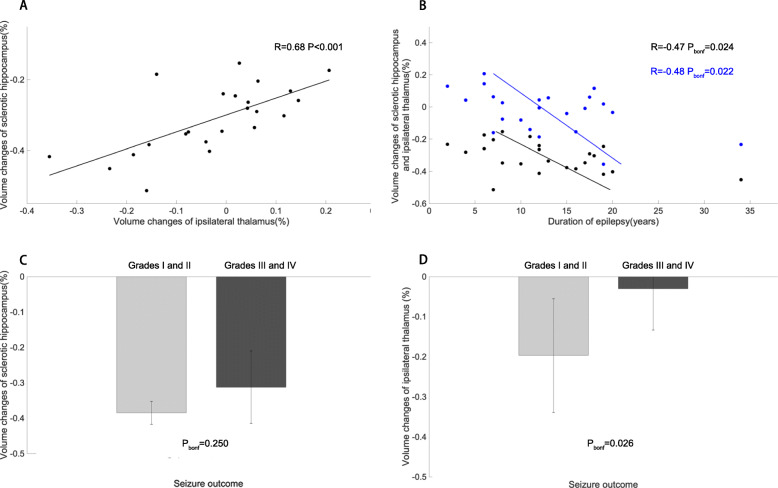
Relationship between volume change of hippocampus and thalamus and clinical variables in patients’ group. (A) Volume change of hippocampus are positively correlated with that of thalamus. (B) Volume changes of hippocampus and thalamus are significantly correlated with duration of epilepsy. (C, D) Volume changes of hippocampus and thalamus are compared between two prognostic groups, respectively. Volume change of hippocampus: (epileptogenic -contralateral) /contralateral volumes. Volume change of thalamus: (epileptogenic -contralateral) /contralateral volumes

Correlation analysis indicated that volume change of the hippocampus was positively correlated with that of the thalamus (R = 0.68, *P* < 0.001, Fig. 4A). Furthermore, volume changes of the hippocampus and thalamus were significantly correlated with duration of epilepsy, respectively (hippocampus: R = − 0.47, P_bonf_ = 0.024; thalamus: R = − 0.48, P_bonf_ = 0.022, Fig. 4B). As shown in Fig. 4, volume change of the thalamus on the ipsilateral side with HS confirmed a marginal significant difference in two groups with different prognoses, however, with regard to that of the hippocampus, there was no significant difference (hippocampus: F = 1.42, P_bonf_ = 0.250; thalamus: F = 5.98, P_bonf_ = 0.026).

## Discussion

Using quantitative measurements, the present study indicated a significant volume decrease in sclerotic hippocampus in patients with MTLE. Intriguingly, a vertex study confirmed that regional contractions were mainly located in the bilateral hippocampus in MTLE groups, and only small patches of shrinkage were noted in the bilateral thalamus. Although thalamic volume on the lesion side was not significantly reduced, volume change of the hippocampus was positively correlated with that of the thalamus. Furthermore, volume changes of the hippocampus and thalamus were significantly correlated with the duration of epilepsy, respectively. Unfortunately, we found a possible difference only in volume change of the thalamus between two groups with different postoperative outcomes in MTLE.

In the present study, hippocampal and thalamic volumes were computed automatically using FreeSurfer software, which provides direct quantitative information and has been validated with high accuracy compared with other methods [19, 20]. Moreover, we revealed the regional alteration of group characteristics using vertex analysis. This approach has been used widely in the study of ageing-related structural alterations, mental disease, dementia, and neurodegenerative disease [17, 18, 21, 22].

Compared with the contralateral hippocampus in patients and the bilateral hippocampus in controls, we only detected a significant decrease in hippocampus volume on the epileptogenic side. This observation was consistent with prior findings [11, 12, 23]. In addition, the specific area of hippocampal atrophy was highlighted by using vertex analysis. Our data showed that inward surface deflation was mainly located in the anterior part of the hippocampus on the ipsilateral side with HS, and was consistent with abnormal hippocampal structure on the epileptogenic side as found in previous studies [2]. Intriguingly, volume and shape changes were not consistent in the contralateral hippocampal or bilateral thalamus. There was no significant reduction in the contralateral hippocampal volume; however, vertex analysis discerned obvious atrophy in patients. Although no obvious sclerosis was found by visual inspection, the contralateral hippocampus was actually affected by the disease. The same was also found in the bilateral thalamus, but the shape of the bilateral thalamus was less affected than that of the hippocampus. So, ipsilateral thalamus was possibly related to sclerotic hippocampus in patients’ group [10–14, 24]. Additionally, this connection might also exist between the hippocampus and thalamus on the contralateral side, but it required further confirmation. The possible reason for this discrepancy may be that regional shape changes are more sensitive to the effects of epileptic discharges, while the global volumes are not significantly affected in these subcortical structures.

In the present, distinct shape patterns of hippocampus and thalamus provide a potential relationship between hippocampus and thalamus in patients with MTLE. We further confirm this opinion by finding the correlation between volume changes of the hippocampus and thalamus on the ipsilateral side with HS. As part of the Papez circuit, the anterior nucleus of the thalamus has been well-documented to interconnect with the ipsilateral hippocampus [16]. The medial thalamus has also been recognized to contribute to forming integrated neural networks of cognition together with other brain structures [25]. In the context of the epileptogenic hippocampus, morphological alterations of specific regions in the thalamus are assumed to result from epileptic discharges spreading into hippocampal-thalamic loops. In addition, considering the high prevalence of memory deficits in patients with MTLE, these results contribute to explaining the impact on memory and other cognitive functions.

We further analyzed the relationship between volume changes of the hippocampus and thalamus with clinical features in this study, rather than only researching the volumes in previous literature [11, 23, 26]. We considered the clinical features of MTLE to be more related to volume changes than to volumes and to more truly reflect the relationship between them. The correlations between volumetric alterations of the hippocampus or thalamus and disease progression were somewhat consistent with previous results, which reported that longer durations were associated with volume reduction of the ipsilateral hippocampus and a decrease in connectivity diversity using predictive models and brain networks analysis [26, 27]. Our study suggested a trend that volume reduction of the hippocampus and thalamus gradually progressed with the duration of disease in MTLE. Furthermore, functional and morphological changes in the hippocampus and thalamus can also affect surgical success and failure after epilepsy surgery [28–31]. Functional abnormalities in MTLE have been described in the thalamus using resting-state functional connectivity analysis and support vector machine (SVM) learning [28]. The thalamus has been involved as a specific nodal hubness in patients who are not seizure-free relative to seizure-free patients and healthy controls [28]. Previous metabolic studies have reported disturbances in PET in MTLE [15, 29]. However, our data did not completely repeat the results of previous studies [30, 31], revealing possible dependence between volume change of the thalamus and postoperative outcomes in patients. Therefore, one possible explanation is that epileptic discharges and transmission may relate to volume loss of the hippocampus and thalamus, which may not affect postsurgical seizure outcomes significantly.

Of note, deep brain stimulation has emerged as a viable therapy in recent years for patients with drug-resistant epilepsy who are not candidates for resection surgery due to various reasons [16]. In particular, a series of clinical trials has shown electrical stimulation of the thalamic nucleus, including the anterior nucleus, to be effective in refractory patients [12, 16, 32–34]. These results reflected the critical role of the thalamus in modulating seizures. However, clinical responses to stimulation of thalamic sub-regions were variable and individualized despite encouraging results. Stimulus targets still need to be refined to improve seizure outcomes. The current study is of great significance for refining targets for neuromodulation.

This study has several limitations. (1) The MTLE samples were relatively small, and our patients were only recruited prospectively from an epilepsy center where they all had preoperative evaluation. There was a possible selection bias that could affect the results of data analysis. Moreover, we hope that more patients from multicenters will be included in future work. (2) In our study, patients with MTLE lacked comprehensive neuropsychological assessment and mental state examination. Therefore, it remains speculative as to the relationship between morphological alterations of the hippocampus and thalamus and emotional and cognitive abilities in patients with MTLE. We only researched the characteristics of the hippocampus and thalamus in patients with MTLE using a cross-sectional study, and therefore we will investigate the relationship between morphological and metabolic characteristics and disease progression using a longitudinal section design in the future.

## Conclusion

We demonstrated the morphological characteristics of the hippocampus and thalamus in mesial temporal lobe epilepsy, showing that shape change was not only found in the sclerotic hippocampus but also in the contralateral hippocampus and bilateral thalamus, and volume changes of the ipsilateral thalamus were related to that of the sclerotic hippocampus, providing new insights into the interrelated mechanisms between the hippocampus and thalamus. In particular, our observations also have potential clinical significance to refine neuromodulated targets.

## Data Availability

The datasets generated and/or analyzed during the current study are not publicly available because they contain privacy information of clinical data. However, they are available from the corresponding author upon request.

## Abbreviations

MTLE: Mesial temporal lobe epilepsy
HS: Hippocampal sclerosis
ET: Emotional seizure
ATM: Automatisms
BA: Behavior arrest
CN: Cognitive seizure
AN: Autonomic seizure
BTC: Bilateral tonic-clonic seizures
ATL: Standard anterior temporal lobectomy
SVM: Support vector machine
